# Modern Bioimaging Techniques for Elemental Tissue Analysis: Key Parameters, Challenges and Medical Impact

**DOI:** 10.3390/molecules30132864

**Published:** 2025-07-05

**Authors:** Jan Sawicki, Marcin Feldo, Agnieszka Skalska-Kamińska, Ireneusz Sowa

**Affiliations:** 1 Department of Analytical Chemistry, Medical University of Lublin, Aleje Raclawickie 1, 20-059 Lublin, Poland; agnieszka.skalska-kaminska@umlub.pl (A.S.-K.); i.sowa@umlub.pl (I.S.); 2 Department of Vascular Surgery, Medical University of Lublin, Staszica 11 St., 20-081 Lublin, Poland; martinf@interia.pl

**Keywords:** LIBS, LA-ICP-MS, XRF, XANES, SEM-EDS, TEM-EDS, bioimaging, human tissues, validation, mapping, quantitative imaging, speciation, LOD, LOQ, selectivity, accuracy, calibration

## Abstract

(1) Background: Elemental imaging methods such as XRF, SEM/TEM-EDS, LIBS and LA-ICP-MS are widely used in clinical diagnostics. Based on the results obtained, it is possible to assess the safety of both standard and innovative therapies, diagnose diseases, detect pathogens or determine intracellular processes. In addition to bioimaging, these techniques are used for semi-quantitative and quantitative analyses. Some of them also enable highly valuable speciation of analytes. However, the quality of information about elemental tissue composition depends on a number of different factors. Although the crucial parameters of quantitative analysis are the same for each technique, their impact varies depending on the bioimaging method. Due to the fact that imaging results are often crucial in clinical decision-making, it is important to clearly indicate and describe the parameters affecting the quality of results in each technique. Therefore, the aim of this review is to describe the influence of these crucial parameters on bioimaging results based on the methodology and results of studies published in the last ten years. (2) Methods: In order to collect relevant publications, the Scopus database was searched using the keywords “element AND imaging AND human tissue”. Next, studies were selected in which methodological aspects allowed relevant conclusions to be made regarding the quality of the results obtained. (3) Results: One of the most important parameters for all techniques is measurement selectivity resulting from the complexity of human tissue. Quantitative analyses using bioimaging techniques are difficult due to the lack of suitable calibration materials. For the same reason, it is challenging to assess the accuracy of the results obtained. Particular attention should be paid to the results obtained for trace elements. (4) Conclusions: The discussed bioimaging techniques are a powerful tool in the elemental analysis of human tissues. Nevertheless, in order to obtain reliable results, a number of factors influencing the measurements must be taken into account.

## 1. Introduction

**Figure 1:**
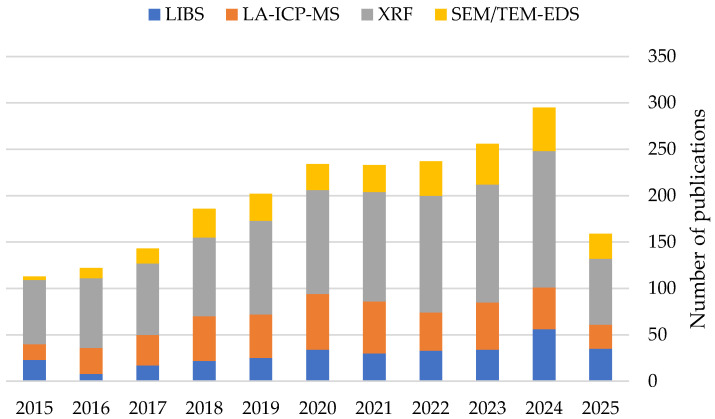
The order of validation and its influence on the quality of measurements.

Elemental analysis of human tissues is crucial for both clinical and environmental health research [1,2,3]. Common techniques include inductively coupled plasma–mass spectrometry (ICP-MS), inductively coupled plasma optical emission spectroscopy (ICP-OES) and, historically the oldest technique, atomic absorption spectrometry (AAS) [1,2]. These techniques require tissue samples to be prepared by digestion in a suitable solution, chemical extraction, or enzymatic or thermal decomposition. It is worth noting that this step increases the time from sampling to results and can also introduce significant errors [4]. The most important of these are loss of analyte and contamination caused by reagents and equipment [4]. These phenomena can occur during both sample preparation and storage [4]. In contrast, this type of specific sample preparation procedure is not required for bioimaging techniques [1]. Bioimaging not only provides information about the distribution of elements in a given structure, but also often about their chemical form [3]. Determining the concentration of essential and toxic elements is important for monitoring and diagnosing diseases, detecting pathogens and for use in many medical fields, including oncology, neurology, surgery, orthopaedics, dentistry, forensic medicine, urology and gynaecology [5,6,7,8,9,10,11,12,13,14,15]. These techniques are also used to analyse nanomaterials with potential medical applications and biomolecules, design innovative bioassays and evaluate intracellular processes [2,3]. Therefore, modern imaging techniques such as laser ablation inductively coupled plasma mass spectrometry (LA-ICP-MS), laser-induced breakdown spectroscopy (LIBS), X-ray fluorescence (XRF) and electron microscopy methods like scanning electron microscopy (SEM) and transmission electron microscopy (TEM) are being increasingly used for the elemental analysis of human tissues [1,2,3] (Figure 1). The widespread use of these techniques means that the results of analyses are described in different ways, such as elemental bioimaging, elemental mapping or multi-elemental imaging [6,8,16,17,18,19,20,21,22,23,24,25]. It is worth emphasising that both trace and macro-elements are the subject of research [5,7,25,26]. These techniques provide key information on therapy safety, the necessity for implant revision and the relationship between diseases and elemental composition [15]. It is also worth noting that the results of these analyses often have a decisive influence on subsequent treatment phases [27,28].

Therefore, this study aimed to review the recent literature and identify key parameters affecting the quality of human tissue imaging techniques. Although these parameters do not limit their applicability, they significantly influence result quality and, by extension, medical decision-making. A comprehensive analysis of this work could reveal the potential future applications of imaging techniques in medicine, as well as their limitations. To gather relevant scientific reports, the term “element AND imaging AND human tissue” was searched in the Scopus database. From the resulting records, manuscripts published no earlier than 2014 that corresponded to the present imaging-based study were chosen, and then the references therein were carefully analysed for additional corresponding papers. We gathered and analysed 37 such publications, whose relevant information is provided. Another important objective of this study was to present these parameters in a way that is accessible to researchers not engaged in chemical analysis, but interested in the subject or seeking potential scientific collaborations.

## 2. Brief Characteristic of Bioimaging Techniques

Laser techniques used in biological tissue analysis include LA-ICP-MS and LIBS. Both techniques utilise laser ablation but they differ significantly in terms of analytical performance and instrumentation.

LIBS involves focusing a high-energy pulsed laser (typically in the nanosecond range) onto the sample surface, which generates a microplasma through rapid ablation of the material. The atoms and ions within the plasma emit characteristic radiation upon relaxation. The emitted light is collected and spectrally resolved to identify and quantify the elemental composition of the sample.

The principle of LIBS can be briefly summarised as follows [29]:

- Heating of the sample surface by laser;
- Ablation of sample surface components;
- Laser-induced creation of microplasma;
- Dissociation (break down) of ablated materials into ions and atoms at excited state;
- Emission of electromagnetic radiation (in two steps: first, continuously; and second, radiation characteristic of the elements of the ablated sample).

The principle of LA-ICP-MS with the relevant figure is presented by Becker et al. in the review paper on elemental bioimaging in samples of various origins [30]. For this technique, a pulsed laser ablates materials from the sample surface, generating a fine aerosol that is transported by a carrier gas (usually argon) into an inductively coupled plasma (ICP) source. The aerosol is atomized and ionised in the plasma, and the resulting ions are introduced into a mass spectrometer for elemental analysis with high sensitivity and resolution.

In both techniques, the laser serves to ablate analytes from the sample surface. In LIBS, the laser excites the analytes, causing them to emit characteristic electromagnetic radiation. In contrast, LA-ICP-MS uses the laser primarily for ionisation, with elemental composition determined from the mass spectrum (abundance vs. *m*/*z*), rather than emission wavelengths as in LIBS.

X-ray techniques are typically divided into three groups: absorption (XAS, XAFS, XANES), fluorescence (XRF, SRXRF) and energy-dispersive methods (EDS) combined with SEM or TEM. X-ray radiation ionises the sample surface, producing complex spectra. The absorption region includes the edge (within ~50 eV, covering pre-edge and rising edge) and the post-edge region (up to ~1000 eV above the edge) [31]. XANES focuses on the edge region, while XAFS extends beyond it; low analyte concentrations may limit measurements to XANES [24]. Both are classified as X-ray absorption spectroscopy. SEM/TEM-EDS and XRF provide comparable results, though they differ in excitation source—electrons in SEM-EDS vs. X-rays in XRF. Theoretical differences are illustrated in Bauer et al. [5], and a summary is shown in Table 1.

## 3. Parameters Relevant to the Quality of Measurements

It is crucial that the obtained determination results are as reliable as possible. To achieve this, all factors that may cause deviations in the measurement results must be identified and their significance assessed [15]. Irrespective of the technique used, the parameters of quantification can generally be described as selectivity, limits of detection and quantification, linearity and measuring range, precision or accuracy [11,13,15,32,33]. The complication is that these parameters can be determined in different ways, depending on the specifications of the analytical method [34]. Over recent years, several papers have been published, the authors of which describe the relevant parameters for the methods.

### 3.1. Selectivity of Measurements

The first important parameter is the selectivity of measurements because it determines the method’s ability to accurately identify and quantify a specific element in the presence of other substances [35]. Although LIBS and LA-ICP-MS both use lasers for ablation, they differ in signal detection—electromagnetic radiation in LIBS vs. ions in LA-ICP-MS—affecting selectivity. In LIBS, emitted signals may overlap with matrix components, while LA-ICP-MS is prone to isobaric, polyatomic and doubly charged ion interferences [11,15].

The influence of polyatomic interference can be determined by performing measurements for standard solutions or special standard reference glasses [13,36]. Bonta et al. present a table with potential polyatomic interferences for various isotopes of Mg, Fe, Ni, Cu, and Zn as well as ^23^Na, ^39^K and ^55^Mn [17]. Hachmöller et al. also note the possibility of interference by ^40^Ar^16^O^+^ ion with ^56^Fe measurements [16]. One possible means of controlling plasma robustness along with its ability to decompose the sample matrix, and therefore polyatomic interferences, is through the daily monitoring of the ^140^Ce^16^O/^140^Ce ratio for a standard solution or ^232^Th^16^O/^232^Th measurements in a standard reference glass material [13,36]. Another approach to omit polyatomic interference is choosing two (or more) isotopes for measurement [17]. Unfortunately, this is not possible for all elements, as some of them naturally form only one isotope (e.g., ^23^Na or ^55^Mn). The advantages of performing measurements for different isotopes are perfectly illustrated in the figure presented by Sajnóg et al. [34]. Although the figure presents isobaric interference, it is evident that ^48^Ti measurements can be affected by the ^48^Ca isotope, in contrast to ^49^Ti [34]. In parallel with isobaric and polyatomic interferences, doubly charged ions may also appear, the control of which is possible by measuring the ^137^Ba^++^/^137^Ba^+^ or ^42^Ca^++^/^42^Ca^+^ ratio [13,36].

In LIBS, selectivity is influenced by different types of interference. Optimising parameters such as laser energy, beam diameter and especially time delay is essential [15]. A properly set delay enhances the detection of second-step element-specific emission and reduces background from first-step continuous radiation [15]. A larger laser spot increases the contribution of the matrix or analyte signals, depending on surface distribution.

Considering selectivity, the most useful information is provided by studies in which several techniques are used to analyse human tissue samples. Therefore, it is worth noting that in parallel to LA-ICP-MS and LIBS, the authors also use other techniques in their analysis, such as ICP-MS, hydrophilic interaction liquid chromatography ICP-MS (HILIC-ICP-MS) or ICP-OES [11,15,17,32,34]. The simultaneous use of LA-ICP-MS and ICP-MS combined with chromatographic separation is particularly noteworthy due to the possibility of analyte speciation.

Both laser-based and X-ray techniques enable tissue bioimaging, but only X-ray methods allow for speciation analysis and provide a range of important clinical information [7]. XRF provides elemental maps, which can be followed by point XANES analysis to assess oxidation states and the analyte’s chemical environment, enabling detailed speciation [6,7]. A meaningful XANES spectrum requires a sufficiently high signal-to-noise ratio [6]. XRF analyses of human tissues are carried out at different resolutions: μXRF, from 1 to 20 µm; sub-μXRF, tenths of μm; and nXRF, from 30 to 250 nm [6,7,18]. Depending on the map size and resolution chosen, different information about the analysed tissues can be obtained. μXRF image analysis performed by Morrell et al. revealed areas of intense cobalt signals with co-located chromium [6]. Sub-μXRF analysis of the same sample also found high signals for chromium and cobalt, except that chromium was present without its Co counterpart, which in turn proved variability in the Co:Cr ratio at a sub-micron level [6]. Further nano-XRF analysis confirmed that the distribution of cobalt and chromium was similar to that obtained with sub-micron resolution [6].

In terms of selectivity, EDS measurements are strongly influenced by surface topography, so a flat, uniform sample is required, which is often difficult to achieve with biological tissues [5]. The emitted X-rays are susceptible to absorption effects related to the surface, and the effective penetration depth in SEM-EDS ranges from 0.5 to 3 µm depending on the composition of the sample [5]. By contrast, XRF enables deeper penetration (up to several millimetres), though this is limited by source intensity and elemental properties [5]. SEM-EDS uses a finely focused electron beam to provide spatial resolution down to the nanometre scale, which can exceed that of conventional XRF by up to three orders of magnitude. By contrast, XRF relies on X-ray excitation and is typically limited to a resolution of a few micrometres [5]. Higher resolution in SEM-EDS reduces sensitivity and increases measurement time, as shown by Bauer et al. in tooth sample analyses [5]. SEM-EDS mapping erroneously suggests the presence of zirconia in the tooth tissue due to the overlap of Zr and P signals (P as a component of tooth tissue) [5]. The greater tissue penetration of excitation radiation in XRF gives information on the average distribution of Ca, P and Ba from a sample volume approximately 500 times larger than that of SEM-EDS [5]. For heavier elements, this volume is even higher, giving clearer maps of distribution for Zn, Zr and W [5]. At the same time, the authors confirm that Zr is a component of the cement and not of the tooth tissue as could be erroneously inferred from the SEM-EDS image [5]. It is worth noting at this point that the differences are directly related to the power of the excitation source. In the case of SEM-EDS, the energy is lower and a Zr signal is observed at a lower energy (~2 keV), which at the same time coincides with the P signal [5]. The XRF excitation source power is greater, and measurement is performed for the Zr signal at ~15.8 keV and is not cross-correlated with other elements. This implies the possibility of spectral interferences during SEM-EDS measurements.

SEM-EDS requires vacuum conditions, necessitating sample dehydration to prevent surface evaporation [5]. Coating (e.g., with carbon) can further reduce evaporation, as applied in THA peri-implant tissue analysis [37]. In contrast, XRF can operate under ambient conditions, particularly for heavier elements [5]. The XRF techniques discussed here use synchrotron radiation sources, offering superior beam energy, resolution and minimal sample damage—enabling further analyses such as tissue structure or cell identification [6,18,19,20,21]. However, tissue dehydration or cryogenic conditions may still be required [6]. The two techniques are complementary: SEM-EDS is better suited for light elements (Z ≤ 12), while XRF is preferred for heavier ones. With specialised detectors, SEM-EDS can also detect elements from boron (Z = 5) upwards [5,38]. Unlike EDS, XRF can be followed by XANES for speciation analysis, though SEM can be coupled with XRD to determine its crystalline structure [22,39]. Table 2 summarises the various factors that influence the selectivity of each technique.

### 3.2. Linearity and Accuracy

Quantitative analysis requires the establishment of a linear relationship between signal intensity and analyte concentration, which is usually achieved through external calibration [35]. In bioimaging, however, this can be difficult due to the solid state and heterogeneity of tissue samples. Gelatine-based standards with known analyte concentrations are therefore commonly used [11,16]. Internal standard calibration (ISC) is also applied to correct for matrix effects using a compound that exhibits behaviour similar to that of the analyte. In tissue analysis, endogenous carbon often fulfils this role, although this does not always ensure accurate results.

Accuracy (sometimes described as trueness) reflects systematic errors (bias) that cause results to differ from the true value. It is assessed using certified reference materials with a known analyte content, usually with uncertainty assigned [35]. However, suitable CRMs for tissue bioimaging are lacking, so materials intended for techniques such as ICP-MS, ICP-OES or AAS are often used instead [6,13,15,21].

Quantification by LA-ICP-MS is referred to as quantitative or elemental bioimaging, or elemental identification [16,17,24,40]. Element distribution maps are generated by scanning parallel lines across the sample surface [13], and integration with an optical stereomicroscope enables real-time viewing with a spatial resolution of up to 1 µm [13,16]. Unlike solution-based ICP-MS, LA-ICP-MS calibration is more complex. As Walas et al. reviewed [41], it often involves matrix-matched gelatine standards or internal standards when blank tissue homogenates are unavailable [16]. Sajnóg et al. applied such a calibration method to analyse oral mucosa tissue [13]. Due to surface inhomogeneity, internal calibration is often utilised to enhance linearity. For instance, Birka et al. employed ^103^Rh and ^115^In for gelatine and skin biopsy samples, respectively, whereas Hachmöller et al. utilised ^103^Rh in liver tissue analysis [11,16]. A summary of internal standards is provided in Table 3.

In LA-ICP-MS, internal standards can include endogenous elements (e.g., C, S), added standards (e.g., Rh, In) or metallic coatings (e.g., ^197^Au) acting as pseudo-IS. The choice of IS can substantially impact the results obtained. Konz et al. reported that using ^13^C instead of sputtered ^197^Au under identical conditions led to signal differences of one order of magnitude for ^24^Mg, 1.5 orders of magnitude for ^56^Fe and two orders of magnitude for ^63^Cu [42]. These variations were not attributed to selective Au adsorption, which was ruled out experimentally, but rather to differences in the ablation process and IS stability [42]. While ^197^Au coatings provide a consistent signal due to their homogeneity, ^13^C varies with sample hydration and cell density, thereby affecting the analyte/internal standard (IS) ratio. Stable IS such as ^197^Au also helps to reduce the effects of instrumental drift during long acquisitions of around 15 h [42]. However, even Au-based IS requires optimisation for tissue thickness. Despite not being recommended, ^13^C is still used in some studies [12,26,32]. Sulphur (^34^S), which is present in amino acids, is another IS option; the stability of the ^33^S/^34^S ratio may confirm the absence of interference [13,32,34].

Moreover, it is assumed that the larger this diameter, the greater the amount of analyte transported to the mass detector. Therefore, some authors normalise the analyte signal against the beam area. As demonstrated by Bonta et al., an increase in ablative surface area results in an increase in matrix effects and delayed ionisation of the analyte, and therefore, a decrease in the normalised signal [36]. The same effect is observed for the pseudo-internal standard sputtered on the sample. Therefore, the normalisation of the analyte signal against this pseudo-internal standard compensates for the matrix effects [36].

Some authors introduce indium along with the ablated material into the detector to monitor the sensitivity of the registered signals or to correct analyte signals due to differences in liquid application during the preparation of dried-droplet calibration standards [11,17]. Gallium was also used to monitor stability of measurements [16]. A glass standard reference material is also used to obtain maximum signal intensity [13].

Gondal et al. used both LIBS and ICP-OES to analyse colon tissues (cancerous and healthy). In their work, Gondal et al. propose a solution (in the form of relevant calculations) to enable the quantitative analysis of LIBS without the necessity of classical calibration (calibration free LIBS–CF-LIBS) [15]. In this application, ICP-OES was used to verify the results of quantitative elemental analysis obtained by the CF-LIBS [15]. The relative accuracy values determined were within acceptable ranges for instrumental analysis [15].

XRF analysis results are often described as mapping [6,19,20,21], bioimaging [43] or simply distribution [44], while the semi-quantitative analysis of the images obtained is referred to as the mass fraction [6,18,21] or particle density [7,8]. The simplest approach is to use only the number of counts of the analytes’ signals, but this does not consider the fluorescence attenuation throughout the sample or X-ray excitation efficiencies [6]. Morrell et al. even refer to such an approach as qualitative, showing significant differences in the Co:Cr ratio compared to an approach that considers these factors, whereby the results obtained are described as semi-quantitative [6]. Semi-quantitative analysis was performed by comparing the spatial mass fraction of the analyte to the background level, defined as the 99.9th percentile of pixel intensities from control samples. The results were visualised as elemental distribution maps, and the proportion of pixels exceeding the background level was reported, along with the maximum local mass fractions. However, this approach is not a typical method for determining linearity and should therefore be considered as semi-quantitative. Similarly, normalisation and deconvolution of XRF spectra with software only allow us to determine the relative concentrations of elements, making it a semi-quantitative analysis [7]. Another approach uses the preparation of several tissue samples embedded on carbon, in which the analyte concentrations increase [21]. With this approach, it is possible to plot a typical calibration curve [21]. Hahn et al. quantify Co and Cr in this way only for mineralised bone tissue samples [21]. Due to the inhomogeneity of bone marrow samples, it was not possible to obtain reproducible Co and Cr contents [21]. Like selectivity, quantitative analysis depends on the measurement resolution used. Analysing synovial sheath tissues at a micro resolution, Morrell et al. determined the percentage coverage for Co and Cr to be 16.4% and 26.4%, respectively, while at sub-micron resolution, these values were 39.8% and 56.8% [6].

During LA-ICP-MS imaging, elements such as P, S, C, Na or K can be mapped simultaneously. This allows for the correlation of analyte measurements with morphological structures or their visualisation [11,17,36]. Moncayo et al. indicate the compatibility of LIBS elemental tissue mapping images with haematoxylin and eosin (HES) staining, allowing the superimposition of LIBS-HES images [25]. This was particularly noticeable when comparing healthy and tumour-transformed tissues. Ca, P, Na and Mg were present in both tissue types, with the difference that more P was contained in the cancer cells [14,25]. Similarly with XRF methods, some authors determine the physiological background levels of elements assessed [18]. In addition, performing co-localisation measurements of the analyte with phosphorus and sulphur enables the determination of whether analyte is present in extracellular or cellular structures [18]. Sulphur is the universally accepted indicator for extracellular structures, while phosphorus is more selective for cellular matrices [18]. Considering the possible toxicity of the elements released from the implant, it is also worth determining the degree of migration into the tissues. Such an approach requires the measurement of matrix macro-elements and the establishment of certain threshold values. Considering the calcium content (1–20% and above 20%) Schoon et al. divided the bone trabeculae into edge and core regions [18]. Through this, the authors identified which metals were present in each region and whether there is a correlation between this content and the type of implant [18].

Due to the lack of relevant (certified) reference materials, not many authors specify the accuracy of the results obtained. Nevertheless, some authors for this purpose use properly prepared CRMs dedicated to other analytical techniques. Sajnóg et al. used ERM-BB422 Fish Muscle (in the form of appropriately prepared pellets) to determine the accuracy of Cu, Zn, Ca and Mg determinations, obtaining satisfactory results [13]. Unfortunately, this CRM did not have certified analyte values of interest; therefore, the authors spiked the samples with a standard method, obtaining acceptable recovery values [13].

### 3.3. Limits of Detection and Quantification

The next crucial parameters are the limits of detection (LOD) and quantification (LOQ). Briefly, LOD is the analyte content possible to detect, but not to quantify [35]. LOQ is the lowest content of analyte that could be determined with proper precision and accuracy/trueness [35]. LOD and LOQ are significantly dependant on the noise level of the measurements; however, its determination is challenging. The literature provides many methods of determining LOD and LOQ, which impedes the direct comparison of the values obtained in different works [13,34].

An important aspect of the analytical capabilities of LA-ICP-MS relates to the LOD and LOQ values. It is important to emphasise the differences due to the very nature of determining LOD and LOQ values with the LA-ICP-MS technique. These parameters are matrix-dependent; thus, some authors distinguish between instrumental (IDL/IQL) and method (MDL/MQL) limits of detection and quantification, based on blank and real-matrix samples, respectively [13]. The literature provides various values for these parameters, which are summarised in Table 4.

Table 3 shows significant variation in IQL values, even for similarly prepared samples. In the two manuscripts, Sajnóg et al. used spiked powdered egg white as the matrix-matched standard. The laser and ICP-MS parameters differed slightly, and the IDL values obtained varied from 2 to 10 times between them (0.55 and 1.1 µg/g for ^49^Ti or 0.24 and 2.2 µg/g for ^27^Al). The differences between some values are several orders of magnitude (^24^Mg and ^66^Zn). Lower MDL values were obtained using dried droplet standards, while higher values were observed in complex matrices like bovine muscle. An interesting conclusion can also be drawn from an analysis of the MDL values for ^27^Al, ^49^Ti and ^51^V. In the case of the values given for Al (1.8 and 6.9 µg/g), Ti (1.5 and 8.1 µg/g) and V (0.82 and 4.6 µg/g), the matrix composition was very similar (powdered egg white was used in both cases). Higher MDL values were associated with different laser settings and slight variations in nebuliser gas flow rates. More specifically, they occurred with smaller spot sizes and higher scan rates.

Determining LOD and LOQ values is also difficult with X-ray techniques. Therefore, in most reports, only vague information can be found regarding the fact that the XRF technique offers high sensitivity, but without giving LOD/LOQ-specific values [6,7,19,20]. Nevertheless, Hahn et al. reported LOD values for Cr and Co of 100 ppm (100 mg/kg) but only for samples of mineralized bone tissue [21]. Schreiver et al. note that the ICP-MS analysis of tissue samples did not detect nickel in almost half of the samples, while nano-XRF analysis revealed high concentrations of small Fe-Cr-Ni-based particles [10]. Moreover X-ray devices without a synchrotron radiation source have higher detection limits, which are typically around 100 ppm [19]. Similarly, the use of a multilayer monochromator provides much lower detection limits than double-crystal monochromators [45].

In the case of SEM-EDS, for lighter elements, the LOD values are less than 1000 µg/g; for medium elements, they are around 1000 µg/g; and for heavy elements, they are up to 3000 µg/g. In the case of the XRF technique, LOD values for elements with small atomic numbers are more than 1000 µg/g [46]. The smallest LOD values (about 20 µg/g) are found for elements with atomic numbers in the range of 35–45 and in the range of 70–90 (between 90 and 120 µg/g) [46].

### 3.4. Measurement Uncertainty

The parameters described above are determined in the validation of analytical procedures. As can be concluded, there are a number of parameters that influence the results to a greater or lesser extent. Depending on the impact of a given factor, the results of the quantitative analysis are close to or deviate from the true value. The method that allows for determining the range within which the true value lies involves determining the measurement uncertainty. Regardless of the method of estimating measurement uncertainty, the result is a certain interval around the mean value of the measurement in which, with a given probability, the true value is found. In the context of the quality of the results, this is important as the uncertainty of measurement is assumed to take into account all random and systematic errors of measurement. This yields ranges that are wider than those resulting from the precision (random errors) of the measurements themselves, which may lead to other conclusions. Regarding the uncertainty of measurement of human tissue materials by laser-based techniques, there are only a few authors who provide relevant information. Bonta et al. determined the measurement uncertainty associated with the use of matrix-matched standards for three concentration levels and three tissue types [36]. Nevertheless, the authors do not present specific values for discussion. For the other methods discussed, there is no information on measurement uncertainty.

To summarise the above, the following conclusions can be briefly drawn:

- Lack of or limited selectivity hinders the application of bioimaging techniques for diagnostic purposes;
- The lack of selectivity is related mainly to matrix effects;
- Complicated procedures for determining linearity can often lead to unreliable quantitative analysis results;
- Selectivity and the resulting accuracy can be assessed using appropriate reference materials (limited availability) or using enriched samples;
- The quantitative bioimaging will be more challenging as the amount of analyte decreases and the matrix becomes more complex;
- Both bias and random error are main components of measurement uncertainty.

**Figure 2:**
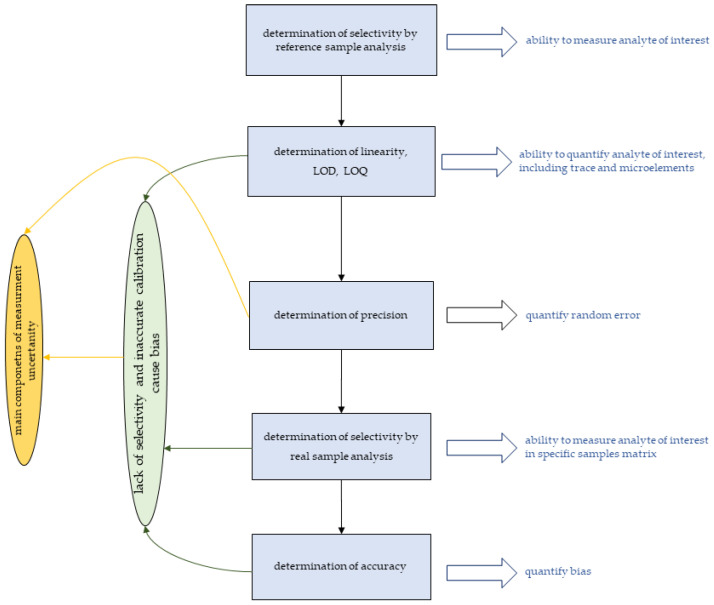
The order of validation and its influence on the quality of measurements.

Considering the nature of all the parameters described, the validation of the measurement method is usually performed in the order shown in Figure 2.

## 4. Application of Bioimaging Techniques in Analysis of Human Tissues

### 4.1. Laser-Based Technique

Several studies have employed laser ablation inductively coupled plasma mass spectrometry (LA-ICP-MS) and laser-induced breakdown spectroscopy (LIBS) to investigate the elemental composition of various human tissues, both healthy and pathological. For instance, the LA-ICP-MS technique was used to analyse peri-implant tissues after spine surgery [47] and oral mucosa tissues for the presence of various elements, including implant components such as Ti, Al, V and S [13,32,34]. In neurodegenerative research, LA-ICP-MS was applied to analyse postmortem brain tissues (white and grey matter and the frontal cortex) of patients with Alzheimer’s disease, identifying P, Fe and C [12]. Similarly, in liver tissues from patients with Wilson’s disease, LA-ICP-MS detected accumulations of Fe, Cu and Ga, and in another study, up to 15 different elements were identified [16,26]. LA-ICP-MS was also employed in cancer research, where mesothelioma tissues were found to contain multiple trace elements such as Au, Pt, In and other transition metals, while tumour tissues from other origins revealed the presence of P, Fe, Cu, Zn and Pt [17,36]. Additionally, Konz et al. used this technique to analyse the elemental composition of human lens tissues from the eye [40].

LIBS has been used as an alternative technique in some cases for multi-elemental imaging of skin tissues affected by cancer, colon tissues from cancer patients and unspecified tumour samples, detecting elements such as Na, K, Ca, Mg, Zn, Cr, Cu, Fe, as well as heavy metals like Hg and Pb [14,15,25]. Bonta et al. used tandem LIBS/LA-ICP-MS to map a tumour-lesioned tissue sample [14]. LA-ICP-MS was employed for the analysis of trace elements, while LIBS was used to analyse minor and major components, including hydrogen and oxygen [14]. The mentioned studies illustrate the broad application of elemental bioimaging in understanding metal accumulation and distribution in human tissues. The details are summarised in Table 5. In 2025, Grenoble University Hospital introduced a specially designed LIBS device for analysing patient biopsies within a hospital environment [48].

An interesting insight into elemental distribution can be gained through the use of complementary analytical techniques. For instance, hydrophilic interaction liquid chromatography (HILIC) with ICP-MS as well as LA-ICP-MS were applied to study gadolinium-based contrast agents (GBCAs) in skin biopsy samples from a patient suspected of having nephrogenic systemic fibrosis (NSF). The use of chromatography enabled the identification of different Gd speciation forms, which would not have been possible using LA-ICP-MS alone. Gd-HP-DO3A was the main GBCA, but analyses have revealed two additional gadolinium-based compounds [11]. Gd-HP-DO3A content in tissue extract was 1.76 ± 0.05 nmol/L which, when converted to gadolinium content in the tissue, gives approximately 0.0046 mg/kg [11]. ICP-MS analysis was also performed to determine the total gadolinium content of the sample [11]. Total mean gadolinium content in patient skin sample was high and varied between 3.02 and 4.58 mg/kg, which confirmed the deposition of this element in the patient’s skin during the course of NSF [11]. Differences between total gadolinium and Gd-HP-DO3A content have been clarified using the LA-ICP-MS technique. Determination of gadolinium and phosphorus by the LA-ICP-MS technique revealed a significant correlation between the contents of these elements, indicating that GdPO_4_ is the dominant form of gadolinium in the skin sample [11].

### 4.2. X-Ray-Based Techniques

Numerous studies in the literature highlight the utility of X-ray-based techniques for high-resolution bioimaging of human tissue samples, enabling detailed elemental analysis. They were used to analyse synovial sheath tissues, peri-implant cancellous bone, periprosthetic tissue, hair, liver, blood, bone and mucosal tissues, periprosthetic bone marrow, breast and ovarian tissue, smears of mucosa, capsular tissue, deep hip tissue granuloma tissue, fallopian tube, uterine horn tissue, red bone marrow, skin and lymphatic tissues, skin and lymph node tissues, synovial fluid, postmortem neuronal, cardiac, hepatic and splenic tissues in various groups of patients (Table 6).

An interesting variant of X-ray-based techniques is nano-XRF. This technique enables highly sensitive and spatially resolved elemental mapping at the nanoscale, making it a powerful tool for investigating the distribution of trace elements in biological tissues. The nano-XRF analysis enabled Schoon et al. to identify the presence of small amounts of zirconium and thallium in a tissue sample from a patient who experienced a severe failure of a revision THA implant [18]. Similarly, Nelson et al. in the nano mode found the presence of trace amounts of As, Pb, Zr, Bi, Ni, Nb and Y in a tissue sample of a titanium-implant patient with peri-implant disease [7]. The same authors confirmed the presence of Zr, Zn, Fe, Hf, Y, Sr and Cr in a sample taken from a patient with zirconia ceramic implant [7].

Depending on the resolution used, various types of information can be obtained through XANES analysis. Point μXANES analysis of synovial sheath tissues conducted by Morrell et al. revealed the presence of chromium in the form of hydrated phosphate (V), while more detailed μXANES mapping enabled the distinction of four different chromium species [6]. Sub-μXANES analysis allowed for the identification of four previously undetermined forms of chromium [6]. It was found that the most abundant form was a combination of CrPO_4_, Cr(OH)_3_ and metallic CoCr [6]. Nano-XANES analysis revealed that cobalt was present in the tissue sample exclusively in metallic form, while chromium species varied depending on the Co:Cr ratio in the analysed area [6].

XANES analysis of periprosthetic hip tissues led Di Laura et al. to conclude that chromium was present in the form of CrPO_4_ and Cr_2_O_3_, while the metallic form was not observed [19]. The same authors identified cobalt(II) in the form of an organic octahedral complex and as a CoCr alloy (Cochrome) [19].

It is worth noting that μXANES analysis can also provide information on the crystalline phase. Schoon et al. clearly confirmed that TiO_2_ in the examined tissue was present in the crystalline form of anatase [18].

Nelson, on the other hand, concluded that titanium was present in one of the samples both in metallic form and as TiO_2_ in the rutile form [7]. Titanium in metallic form, as well as in rutile and anatase phases, is most frequently reported, although there are also cases where amorphous forms of TiO_2_ have been identified [19].

More detailed information on elemental composition can be obtained by combining different techniques, and this approach has been applied in several studies.

Measurements using a combination of the above techniques also provide important information. A combination of LA-ICP-MS and XRF/XANES techniques was reported by Swiatkowska et al. [24]. LA-ICP-MS analysis determined the content (in ug/g) of Co and Cr in cardiac tissue [24]. XRF analysis, on the other hand, made it possible to map the elements in the samples and to perform XANES speciation measurements for hotspots of Cr, Co and Ti [24]. In both cases, the sizes of the maps obtained were similar at 3 × 2 mm and 3 × 3 mm for XRF and LA-ICP-MS, respectively. The authors do not describe the differences in mapping results between the two techniques. At the same time, they emphasise that XRF techniques allow the mapping of large areas, have low LOD values and are not destructive to tissue samples [24]. Disadvantages of the technique include its limited availability, long measurement times and lack of representativeness due to the mapping of selected tissue areas [24].

Schreiver et al. in turn combined the XRF/XANES technique with ICP-MS in the postmortem analysis of tattooed skin and lymphatic tissues [10,23]. In the context of a comparison between the two techniques, the authors conclude that the nickel content was noticeably elevated in only three samples (compared to the controls) [10]. In contrast, XRF analysis showed an abundance of nickel-containing particles in all tissues analysed [10]. Such discrepancies were not found for iron due to its high content in tattoo pigments [10]. At the same time, XANES analysis revealed that nickel was present mainly in metallic form with minor admixtures of sulphate and hydroxide [10]. This clearly confirms that the XRF technique can detect a given element, even in a small area, but that its content in relation to the total tissue analysed is so negligible as to be undetectable by the sensitive ICP-MS technique.

In other reports, the authors use SEM-EDS or TEM-EDS and ICP-MS for human tissue analysis [33,37,51,52,53]. The purpose of the majority of the papers cited was to analyse the different tissues of patients with hip implants and determine the various correlations between the implants and their safety (including elemental content). Therefore, the authors do not concentrate directly on comparing the two methods. Nevertheless, by examining the papers cited, several methodological conclusions can be drawn regarding the use of both methods. Wang et al. analysed metal particles in synovial fluid samples using the SEM-EDS technique, while they used the ICP-MS technique for quantitative analysis of metal ions in centrifuged samples [53]. It is worth noting that different preparations of the same sample combined with analysis by different techniques lead to more informative results. SEM-EDS was also used by De Pasquale et al. to detect particles in synovial fluid and ICP-MS to quantify Co and Cr in serum, except the sample preparation procedures were different [33]. Considering the quality of the results obtained, it is worth noting at this point that De Pasquale et al. used appropriate CRMs to assess the accuracy of the analyses. Inferring from the methodology given, a different assumption in their work was made by Scharf et al. [37]. For the preparation of peri-implant tissue samples for ICP-MS analysis, the authors used aqua regia [37], which led to the assumption that under such conditions, they determined the sum of the elements—both in metallic and ionic form. Babis et al. used the ICP-MS technique to analyse the tantalum content of serum obtained from a patient with non-osseointegrated, severely worn tantalum augmentation [51]. The content of tantalum in serum is of the order of ng/L; therefore, ICP-MS is a technique suitable for the analysis of such low concentrations. At the same time, the authors analysed the peri-implant tissue using the SEM-EDS technique, showing a very high tantalum content [51]. To map gadolinium (a contrast agent) in postmortem neural tissues, McDonald et al. used TEM-EDS [52]. The same samples were also analysed by the ICP-MS technique and revealed a correlation between gadolinium deposition in neuronal tissue and intravenous GBCA exposure [52].

**Table 6:** X-ray techniques in analysis of various human tissues.

Sample	Implant/ Group of Patients	Number of Patients/Samples (Without Control)	Element	Technique	Remark	Reference
lung tissue	healthy and idiopathic pulmonary fibrosis	2	Ca, Zn, S, Fe, Al, Cr, Cu, Ti, Mn, P	XRF nano-XRF (100 nm)	mapping	[54]
tooth	no determinant	3 + additional samples (stored for more than a decade)	Ca, P, Ba, Zn, W, Zr, Sr, Fe	μXRF	elemental maps	[5]
			P, Ca, Ba, Zn, W, Zr	SEM-EDS		
			P, Ca, Ba, Zn, W, Zr	CμXRF		
synovial sheath tissues	hip	2	Co, Cr, Mo	μXRF and μXAFS (3 μm resolution)	mapping	[6]
			Co, Cr (speciation)	sub-μXRF and sub-μXANES (600 nm resolution)	mapping	
			Co, Cr	nano-XRF and nano-XANES (250 nm resolution)	mass fraction	
peri-implant cancellous bone	hip, knee	14	Co, Cr, Ti (main analytes) Fe, S, P, Ca (matrix structures)	μXRF (10, 3 and 2 µm)	mapping/ mass fraction	[18]
			Ti (speciation)	μXANES		
			Ti, Zr, Ta	nano-XRF (60 and 30 nm)	mapping	
tissues	dental	13	Ti, Zr (main analytes) P, S (matrix structures)	μXRF (resolution from 1 to 20 μm)	particle density (mass fractions)	[7]
			Zr, Zn, Fe, Hf, Y, Sr, Cr, Ni, Nb	nano-XRF (60 nm resolution)	particle density (mass fractions)	
			Ti (speciation)	μXANES (between 1 and 10 μm)		
periprosthetic tissue	hip	7	Co, Cr, Ti	μXRF	mapping	[19]
			Co, Cr, Ti (speciation)	µXANES		
hair	no determinant	4	Hg	nXRF (50 nm)	distribution of mercury	[44]
			Hg	XANES (high resolution)	speciation	
liver	hip	1	Co, Cr, Ca	μXRF μXAS	mapping	[20]
blood			Co, Cr	-	quantitatively	
bone and mucosal tissues	dental	12	Ca, Ti, Fe, P	XRF	-	[45]
soft tissue, bone marrow, mineralized bone tissue	hip	13	Co, Cr	μXRF (80 µm resolution)	mapping and mass fraction	[21]
periprosthetic bone marrow	hip, knee	8	Co, Cr and Mo	nano-XRF (60 nm resolution)	bioimaging	[43]
breast, ovarian tissue	various cancers	60 (samples)	Zn, Fe, Cu, Ca	μXRF	mapping particle density	[8]
soft tissues	dental	31/36	C, N, Na, K, O (controls) Ca, P, Ti, Zr, Al, Si, F, Cl, Fe, Zn, Pt, S, Mg, Br, Pb, Ni, Ba, Bi, La	SEM-EDS	mean percentages represent the composition of the elements	[55]
periprosthetic tissue	hip	53	Cr, Co, Mo, Ti, V, Fe, P, O	SEM-EDS TEM-EDS	mapping	
			Cr	XRD	crystalline structures	[22]
blood			Co, Cr	-	quantitatively	
tissue	patients who had tongue and/or lip piercings	16	C, K, Ca, O, Na, Mg, Al, Cr, Mn, Fe, Co, Si, S	SEM-EDS	semi-quantitatively	[56]
smears of mucosa			Ca, C, O, Na, Mg, Al., Mo, Si			
capsular tissue, deep hip tissue granuloma tissue	hip	26	Ti, Cr, Co, Fe, Ca, Mo, C, Cl, Si, P	SEM-EDS	qualitatively	[39]
blood serum			Co, Cr, Ti	-	-	
tissues			Cr, Al	XRD	crystalline structures	
fallopian tube or uterine horn tissue	intrauterine device	10	endogenous particles contain Na, P, S, Ca, Cl, K, Fe, Sn, Si, Al, Ca, Fe, Ti, Sb -based Au, Al, Pt	SEM-EDS	qualitatively	[9,57]
red bone marrow (postmortem)	hip, knee	6	particles of combined Co, Cr, Mo, Fe, Ni, Ti, Al, V	SEM-EDS (resolution from 50 nm to 6 μm)	qualitatively	[58]
skin and lymphatic tissues tattooed skin	tattooed skin (postmortem)	20 (skin) 25 (lymph node)	Br, T, P, Cl, P, S, K, Ca	μXRF (from 0.5 µm to 5 µm)	mapping	[23]
			Ti	μXANES (from 1 µm to 10 µm)	speciation	
skin and lymph node tissues	tattooed skin (postmortem)	5	Fe, Cr, Ni, Ti, Cu	nano-XRF (50 nm)	elemental maps	[10]
			Cr, Ni	XANES	speciation	
periprosthetic tissues	hip	18	Cr, Co, Mo, Si, Ca, P, Na	SEM-EDS	elemental composition	[37]
synovial fluid	hip	40	Co, Cr particles	SEM-EDS	semi quantitatively	[33]
periprosthetic tissue	hip	1	Cr, Co, Ta, C, O, S, Ti, N, Na	SEM-EDS	area fractions	[51]
postmortem neuronal tissues	gadolinium-based contrast agents brain magnetic resonance examinations	13	C, Cs, Cu, Gd, O, Os, Pb, Ti, V	TEM-EDS	distribution	[52]
cardiac, hepatic splenic postmortem tissues	hip, knee	5/13	Co, Cr	LA-ICP-MS	distribution	[24]
			Co, Cr, Ti	μXRF (5, 3 μm)	mapping	
				μXANES (3 μm)	speciation	

The potential capabilities in the analysis of specific tissue types or clinical contexts is briefly summarised in Table 7.

Considering the information summarised in Table 7 and point-conclusions from Section 3, it should be clearly stated that none of the techniques is universal. At the same time, taking the above into consideration, it can be concluded that the most suitable methods for analysing trace elements and microelements will be LA-ICP-MS and LIBS. At the same time, LA-ICP-MS seems to be a better technique due to lower detection limits and better robustness against matrix effects. However, it has lower throughput and higher operating costs. On the other hand, the LIBS technique seem to be the appropriate one in macro-element analysis. For liquid samples such as blood, plasma or serum, both laser-based techniques are suitable, but the XRF technique requires less sample preparation. If the researcher aims to determine the crystal form, the number of bonds or the chemical compound a given element appears in, the only means of achieving this is by using XRF. None of the techniques listed are applicable for in situ analysis of human solid tissues. However, in recent years, procedures have been proposed that enable the analysis of biological fluids using LA-ICP-MS (less than 1 min), XRF or LIBS [59,60,61]. Hence, these techniques are widely used to analyse samples taken during surgery rather than in point-of-care settings.

### 4.3. Alternative Techniques Potentially Applicable to Bioimaging

Among the techniques potentially applicable to bioimaging are TOF-SIMS (Time-of-Flight Secondary Ion Mass Spectrometry) and MALDI-TOF-MS (Matrix-Assisted Laser Desorption/Ionisation Time-of-Flight Mass Spectrometry). While their primary use lies in the analysis of organic compounds, their potential utility in elemental analysis is also worth mentioning [62]. Risseeuw et al. performed TOF-SIMS bioimaging on calcified Bruch’s membrane [63]. They confirmed that the calcifications originated from inorganic hydroxyapatite (HAP), simultaneously mapping organic tissue via proline analysis [63]. Other researchers also performed simultaneous imaging of HAP, lipids and proteins in eye tissues [64,65]. Similarly, Biesemeier et al. used Nano-SIMS to map S, Cu, Ca, Fe and Na, as well as species such as CN^−^ and PO_2^−^_, in perimacular eye tissue [66]. Nano-SIMS and TOF-SIMS is also widely applied for imagining metallodrugs in human cervical [67], ovarian [68], colon [69] and breast [70] cancer cells as well as glioblastoma cells [71,72]. TOF-SIMS was also used to evaluate the effect of metallodrugs on the homeostasis of Fe, Cu and Zn in both cancerous and normal human cells [73]. Similarly, MALDI-TOF-MS was used in the analysis of tumour spheroids [74], colorectal or ovarian tumour samples [75,76], Pt-based drug interactions with two metallothionein protein isoforms [77], or new chemotherapeutics [78,79,80].

In the context of the techniques discussed so far, it is important to emphasise that, while both TOF-SIMS and MALDI-MS allow speciation, only TOF-SIMS enables depth profiling [63,81]. It is equally important to note that Nano-SIMS enables the analysis of both light and most of the heavier elements [82]. MALDI-MS is used to analyse larger organic structures, such as proteins, lipids and organic complexes. However, compared to LA-ICP-MS, for instance, MALDI or Nano-SIMS analysis requires time-consuming sample preparation [62]. At the same time, TOF-SIMS achieves satisfactory sensitivity at the mg–μg/kg level, while MALDI-MS offers even higher sensitivity, reaching the μg/kg level or lower [62]. The spatial resolution is 100–500 nm and 40–50 nm for TOF-SIMS and Nano-SIMS, respectively [62].

## 5. Conclusions

Our study showed that bioimaging techniques, such as LA-ICP-MS, LIBS and X-ray-based methods, are being increasingly applied for the elemental analysis of human tissues. These techniques have been used, among other things, for mapping the spatial distribution of elements in pathological tissues, such as tumours, organs affected by metal-related diseases (e.g., Wilson’s disease), or tissues surrounding medical implants. In addition to mapping, these techniques are also used for quantitative analysis.

One of the main limitations of these techniques is their restricted ability to distinguish between different chemical species (speciation)—particularly in the case of LA-ICP-MS and LIBS, which provides total elemental content but limited chemical form information. Nevertheless, speciation is possible with the XANES technique. Additionally, sample preparation and matrix effects may influence the accuracy of quantitative determinations of all techniques discussed. At the same time, these techniques are also used for quantitative analysis, but this is more challenging and requires the consideration of significantly more factors that may influence the result than in bioimaging.

On the other hand, the main advantages of these techniques include high spatial resolution, multi-elemental detection capabilities and the ability to generate visual elemental distribution maps both in tissues and at the cellular and even sub-cellular level. These features make them particularly valuable for biomedical research, pathology and toxicology.

Looking ahead, future perspectives involve integrating these imaging methods with advanced molecular and omics-based techniques, such as proteomics or metabolomics, as well as improving data analysis with artificial intelligence and machine learning. The latter aspect has shown tremendous potential, where deep learning mechanisms are used in image reconstruction and temporal and spatial resolution enhancement [59,83]. Such advancements will enable deeper insights into disease mechanisms, trace metal homeostasis and the long-term biocompatibility of implanted materials. It is worth emphasising that the bioimaging techniques discussed in this paper are becoming increasingly popular. Nevertheless, other more elaborate bioimaging techniques are also used in this area, such as synchrotron μ-FTIR [23], surface-enhanced Raman scattering [84], matrix-assisted laser desorption/ionisation mass spectrometry imaging [85], high-resolution X-ray ptychography [86] and time-of-flight secondary ion mass spectrometry [87]. The application potential of these techniques is enormous, but they enable the analysis of organic compounds, which was not the subject of this study.

## Figures and Tables

**Table 1:** Comparison of elemental imaging techniques.

Feature	LA-ICP-MS	LIBS	XRF	SEM-EDS	TEM-EDS
Full Name	Laser Ablation Inductively Coupled Plasma Mass Spectrometry	Laser-Induced Breakdown Spectroscopy	X-ray Fluorescence	Scanning Electron Microscopy with Energy-Dispersive X-ray	Transmission Electron Microscopy with Energy-Dispersive X-ray
Spatial Resolution	5–100 µm	~10–100 µm	~0.05–100 µm	~1 µm	<20 nm
Detection Limit (LOD)	µg/kg	mg/kg	mg/kg	Tenths of weight %	Tenths of weight %
Quantification	Yes	Yes	Yes	Semi-quantitative	Semi-quantitative
Sample Destruction *	Semi-non-destructive	Non-/minimally destructive	No	No	No
Suitable Sample State	Solid (flat and polished)	Solid (minimal preparation)	Solid (minimal preparation)	Solid (degreased and dried)	Ultrathin slices (~100–150 nm)
Light Element Detection	Limited	Yes (H, C, N detectable)	Limited	Limited	Limited
Sample Preparation Complexity	Medium (polishing, standards)	Low (clean surface)	Low (minimal preparation)	Medium (mounting, coating with Au/C)	High (FIB, ultramicrotomy, thinning to electron transparency)
Analysis Time	Minutes to hours (mapping)	Seconds to minutes	Seconds to minutes	Minutes per point or map	Long (due to preparation and imaging)

* refers to measurement, not to sample preparation step.

**Table 2:** Factors influencing selectivity.

LA-ICP-MS	LIBS	XRF	SEM-EDS
laser energy ablation area isobaric interferences polyatomic interferences doubly charged ions plasma robustness dwell time instrumental drift gas flows	laser energy ablation area readout time delay surface roughness gate width measurement atmosphere repetition rate	absorption effects power of the excitation source type of radiation source signal intensity for speciation topography of the layer measurement atmosphere	power of the excitation source measurement atmosphere surface topography sample conductivity

**Table 3:** Internal standards for LA-ICP-MS in analysis of various human tissues.

Analyte	Internal Standard	Reference
^157^Gd, ^158^Gd, ^160^Gd, ^31^P, ^44^Ca	^103^Rh, ^115^In	[11]
^54^Fe, ^56^Fe, ^63^Cu, ^65^Cu, ^64^Zn, ^68^Zn	^197^Au (pseudo-internal standard)	[40]
^194^Pt,^195^Pt,^196^Pt, ^13^C, ^31^P, ^34^S	^97^Au (pseudo-internal standard)	[36]
^23^Na, ^24^Mg, ^25^Mg, ^39^K, ^42^Ca, ^44^Ca, ^55^Mn, ^56^Fe, ^57^Fe, ^58^Ni, ^60^Ni, ^63^Cu, ^64^Zn, ^65^Cu, ^66^Zn	^115^In, ^197^Au (pseudo-internal standard)	[17]
^27^Al, ^49^Ti, ^51^V	^34^S	[13]
^54^Fe, ^56^Fe, ^63^Cu, ^65^Cu	^69^Ga, Rh	[16]
Na, Mg, P, K, Ca, Ti, Cr, Ni, Cu, Zn, Pb	C, S	[26]

**Table 4:** Method and instrumental detection/quantification limits in LA-ICP-MS.

Isotope	IDL [µg/g]	IQL [µg/g]	MDL [µg/g]	MQL [µg/g]
^23^Na			5.7 [17]	
^26^Mg	14 [32]		10.6 [17] 419 [32]	
^24^Mg			2.3 [17]	
^27^Al	0.83/0.24 ^1^ [34]	2.5/0.72 ^1^ [34]	4.8/1.8 ^1^ [34]	14/5.3 ^1^ [34]
	2.2 [13]		6.9 [13]	
	4.1 [32]		14 [32]	
^39^K			13.2 [17]	
^43^Ca	450 [32]		1174 [32]	
^49^Ti	0.78/0.55 ^1^ [34]	2.4/1.7 ^1^ [34]	0.84/1.5 ^1^ [34]	2.5/4.4 ^1^ [34]
	1.1 [13]		8.1 [13]	
	14 [32]		21 [32]	
^51^V	0.24/0.1 ^1^ [34]	0.73/0.30 ^1^ [34]	0.58/0.82 ^1^ [34]	1.8/2.5 ^1^ [34]
	0.80 [13]		4.6 [13]	
^55^Mn	1.8 [32]		0.1 [17] 4.7 [32]	
^56^Fe	5 ^2^ [16]	18 ^2^ [16]	0.8 [17]	
^57^Fe	43 [32]		3.4 [17] 98 [32]	
^58^Ni			0.1 [17]	
^60^Ni			0.4 [17]	
^63^Cu	1 ^2^ [16]	4 ^2^ [16]	0.1 [17] 6.5 [32]	
^65^Cu			0.2 [17]	
^64^Zn			0.1 [17]	
^66^Zn	18 [32]		0.2 [17] 61 [32]	
^157^Gd,^158^Gd,^160^Gd	3.0 [11]		9.0 [11]	
^195^Pt			1.6 [36]	

^1^ Dependent on strategy; ^2^ no information of whether an instrumental or methodical limit is given.

**Table 5:** LIBS and LA-ICP-MS in analysis of various human tissues.

Sample	Implant/ Group of Patients	Number of Patients/Samples (Without Control)	Element	Technique	Remark	Reference
gastric cancer cells	human cell line	-	Zn	LA-ICP-MS	35 μm spot size	[49]
skin biopsy samples	nephrogenic systemic fibrosis	1	Gd, Ca, P	LA-ICP-MS	KED ^1^ 50 μm spot size	[11]
oral mucosa tissues	dental	30	Ti, Al, V, S	LA-ICP-MS (quantitatively)	50 μm spot size	[13]
oral mucosa tissues	dental	no information	Ti, Al, Ca, Mg, Zn, Cu, Fe, Mn, S, C	LA-ICP-MS (quantitatively)	25 μm spot size	[32]
oral mucosa tissues	dental	12	Ti, Al, V S, C, Mg, Ca	LA-ICP-MS (quantitatively)	100 μm spot size	[34]
white and grey matter and frontal cortex tissues (postmortem)	Alzheimer’s disease	4	P, Fe, C	LA-ICP-MS (quantitative imaging)	CRC ^2^ 80 × 80 μm laser beam	[12]
liver	Wilson’s disease	3	Fe, Cu, Ga	LA–ICP–MS (elemental bioimaging)	KED ^1^ 100 μm spot size	[16]
tissue	human malignant mesothelioma	1	Na, Mg, K, Ca, Mn, Fe, Ni, Cu, Zn, In, Au	LA-ICP-MS (elemental bioimaging)	40 μm laser diameter	[17]
liver	Wilson’s disease	6	C, Na, Mg, P, S, K, Ca, Ti, Cr, Fe, Mn, Ni, Cu, Zn, Pb	LA-ICP-MS	-	[26]
tissue	human malignant mesothelioma	1	Pt, C, P, S, Au	LA-ICP-MS	50 μm laser diameter	[36]
human lens	eye	5	Fe, Cu, Zn, Au	LA-ICP-MS (quantitative bioimaging)	200 μm	[40]
healthy teeth, deciduous teeth, teeth filled with amalgam and composite restorative materials	-	-	Ca, K, Mg, P, Na, Sr, Cu, Cr, Fe, Ba, Pb, Zn, Hg, Al	LIBS qualitative	-	[50]
tissue	human tumour	1	P, Fe, Cu, Zn, Pt	LA-ICP-MS	mapping 40 μm per pixel	[14]
			C, H, O, Na, K, Ca, Mg	LIBS	-	
skin tissues	various cancers	3	P, Al, Mg, Na, Zn, Si, Fe, Cu, Ca	LIBS (multi-elemental imaging)	-	[25]
colon tissues	colon cancer	15	Pb, Cr, Ce, Hg	LIBS	-	[15]

^1^ Kinetic energy discrimination; ^2^ collision reaction cell.

**Table 7:** Applicability of bioimaging techniques.

Feature	LA-ICP-MS	LIBS	XRF	SEM-EDS	TEM-EDS
Trace element detection	most suitable	suitable	suitable	limited	limited
Macro-elements	limited	suitable	limited	limited	limited
Sample re-analysis	limited	limited	possible	possible	possible
Depth profile analysis	tens of micrometres	<500 μm	several tens of micrometres	few micrometres	limited by sample thickness
Speciation	not possible	not possible	possible	not possible	not possible
Throughput	moderate	high	high	slow	slow
Operating costs/availability	expensive	moderate	moderate	expensive	very expensive
Solid samples	suitable	suitable (solid surfaces)	tissue slices	excellent resolution	excellent resolution
Liquid samples (blood, plasma)	requires dry blood spot or cryogenic ablation	requires drying	plasma, serum with or without dilution; blood need preparation step	difficult due to high vacuum environment	difficult due to high vacuum environment
Relative sensitivity to matrix effects	high (due to fluctuations in composition, structure and moisture content)	very high (due to laser energy absorption, plasma formation)	moderate to high (due to varying density, thickness and water content)	low to moderate (depend on atomic number and competing absorption-fluorescence effects)	low (due to sample thickness)
Linear range (in mg/kg)	~0.01–10,000	~10–10,000	~1–100,000	~100–100,000	~100–50,000
Maximum mapping area	>cm^2^	~cm^2^–several cm^2^	~cm^2^–several cm^2^	~mm^2^	<<mm^2^

## Data Availability

All data are included within the article.
